# Socioeconomic benefits of conserving Iran’s water resources through modifying agricultural practices and water management strategies

**DOI:** 10.1007/s13280-021-01534-w

**Published:** 2021-03-23

**Authors:** Fatemeh Karandish

**Affiliations:** 1 Water Engineering Department, University of Zabol, Zabol, Iran; 2 Multidisciplinary Water Management, Faculty of Engineering Technology, University of Twente, P.O. Box 217, 7500 AE Enschede, the Netherlands

**Keywords:** Blue water scarcity, Food security, Pressurized irrigation, Soil mulching, Water saving irrigation, Yield gap closure

## Abstract

Sustainable development requires modifying the current consumption pattern of natural resources. This study investigates efficient tactics for reducing the unsustainability and inefficiency of human’s food-related blue water consumption alongside improving national environmental and socioeconomic status. As a case study for Iran, 15 alternative management scenarios (AMS) were defined compared to the current on-farm management, and their effects were assessed on a monthly scale. Based on the results, 45.5 billion m^3^ y^−1^ (BCM) blue water is consumed within the croplands, 78% and 34% of which are unsustainable and inefficient, respectively. AMCs reduces the unsustainable and inefficient blue water consumption by 2–17 BCM and 2–13 BCM, respectively. The combination of yield gap closure, drip irrigation, soil mulching, and deficit irrigation has the largest effect on blue water saving; it releases or changes the status of monthly blue water scarcity in 11 provinces; increases field-employees by 132%, food security by 9%, international food-export by 87%, and gross domestic production by 54%. However, it doesn’t fully address blue water overconsumption in the summer period; hence, further measures are needed to reduce blue water scarcity to the sustainable level in these environmental hotspots.

## Introduction

Agriculture is the biggest freshwater user in the world and has the largest contribution of 92% in global blue water consumption (Hoekstra et al. 2012). Continuing the current pattern of agricultural water consumption threats the sustainability of future food security due to water shortage challenges (Steffen et al. 2015; Fitton et al. 2019). Reducing the vulnerability of sustainable crop production to water shortage is, thus, becoming a global concern (Jägermeyr et al. 2015). Hence, many researchers tried to find out pathways to increase food security under water scarcity status.

The opportunity of enhancing crop’s yield, named yield gap closure (YGC), is one of the promising solutions which received large attention by many researchers (e.g., Phalan et al. 2011; Pretty et al 2011; Mueller et al. 2012; Garnett et al. 2013; Davis et al. 2017; Rosa et al. 2018). YGC is achieved through irrigation or agricultural practices and technology improvement. Expanding irrigated lands without overshooting the sustainable blue water availability is only possible in 25% of the global croplands (Rosa et al. 2020). Such expansion may provide 37% more calories, enough to feed 2.8 billion people, at the cost of 408 billion m^3^ year^−1^ extra blue water consumption (48% more than the current condition) (Rosa et al. 2018). Nevertheless, replacing all rainfed lands with irrigated ones require 1130 billion m^3^ y^−1^ additional blue water which is not available in many countries, and therefore, it may result in further unsustainable blue water consumption (Davis et al. 2017), mainly through overexploiting groundwater or fossil water abstraction (Nouri et al. 2019). Hence it is advised to improve crop’s yield through methods which don’t entail extra blue water consumption (Davis et al. 2017). Improving technologies and agricultural practices or modifying crops traits are among such methods (Ali and Talukder 2008; Chukalla et al. 2015; Jägermeyr et al. 2015; Rosa et al. 2018). For instance, YGC could be achieved through a transition toward drip/sprinkler irrigation (Jägermeyr et al. 2015), shifting cropping dates, modifying tillage practices, improving crop’s traits or genetic (e.g., Ali and Talukder 2008; Sinclair and Rufty 2012; Chukalla et al. 2015; Karandish et al. 2017; Darzi-Naftchali and Karandish 2019), and improving nutrient applications or effectively controlling pests, diseases, and weeds in the croplands (Mueller et al. 2012; Sinclair and Rufty 2012). However, the effectiveness of these practices is site-specific and need to be investigated from place to place.

Lessening agricultural blue water consumption is the other solution to this concern; it can reduce blue water scarcity and, consequently, increases food security. In this regard, earlier researchers proposed various methods to reduce applied water at the field scale including efficient irrigation techniques such as drip/sprinkler irrigation (Postel et al. 2001; Nouri et al. 2019), precision, and on-demand irrigation techniques (Greenwood et al. 2010), water saving irrigation strategies such as deficit irrigation (Chai et al. 2016; Karandish 2016), partial-root-zone drying (Karandish and Šimůnek 2018, 2019), and supplemental irrigation (Chukalla et al. 2015), soil mulching for reducing non-productive water demand (Chukalla et al. 2015; Pi et al. 2017; Nouri et al. 2019), conservation tillage practices (Liu et al. 2013) or soil organic amendments application (Miller and Naeth 2019) for improving soil’s water holding capacity and properties, crop diversification (Lin 2011) and rotation (EIP-AGRI 2016) for enhancing the resilience of crop production to water shortage, cultivating more drought-tolerant crop cultivars to reduce the risk of production loss under water stresses (Hu and Xiong 2014), or reducing croplands under water intensive crops (Chouchane et al. 2020; Karandish et al. 2020). The effect of these practices may also vary from place to place, and from crop to crop, and therefore, this requires to be investigated locally.

In this research, we improve upon previous studies by evaluating the individual or simultaneous effects of YGC and improving agricultural and water management practices on alleviating water scarcity. We did our assessment at the national scale in contrast to the other studies that mainly focused on the field-scale consequences. We built our comparative assessment based on the water footprint concept (Hoekstra et al. 2011). Fifteen alternative management scenarios were defined compared to the base case (i.e., the current condition), and their consequences were assessed in terms of their effects on inefficient and unsustainable blue WF, and blue water scarcity index (BWS). While some researchers tried to quantify further blue water requirement under their proposed management scenarios (Chukalla et al. 2015; Davis et al. 2017; Rosa et al. 2018; Nouri et al. 2019), they rarely address the impacts of these scenarios on the efficiency and sustainably of blue WF. In addition, we did our BWS evaluations on a monthly scale in contrast to other studies that did on an annual basis (Vörösmarty et al. 2000; Oki et al. 2001). The annual assessment may provide an underestimation of environmental flow requirements (EFR) violation since it ignores intra-annual variabilities. We also did our monthly BWS assessment at the province scale to reveal the spatial variations as well.

We also improved upon earlier studies by assessing the socioeconomic consequences beside the environmental ones. We quantified the effects of implementing the best defined scenario on the number of farm-employees, provincial food security, the fraction of international food export, and gross domestic production. While such analyses may increase the acceptability of the proposed strategies, it is almost ignored in earlier studies that focused on water-related issues.

## Materials and methods

### Main definition and assumptions

We built our study on the basis of the WF assessment (Hoekstra et al. 2011). WF is a multi-dimensional indicator of water consumption which quantifies an appropriation of water resources along entire value chains (Hoekstra et al. 2011). It comprises of three main components: green, blue, and gray WFs (Hoekstra 2019; Hoekstra et al. 2011). We assessed Iran’s agricultural WF and formulated the response options based on three indices: unsustainable EF, inefficient WF, and BWS. Following earlier researches (Hoekstra et al. 2011; Madani and Khatami 2015; Mekonnen and Hoekstra 2012, 2016; Hoekstra 2019), the standard definitions of the terms and relevant assumptions used in this study are summarized in Table 1.

**Table 1:** Main definitions of the terms applied in the current research for the agricultural sector

Term	Symbology	Definition and assumptions	Source of definition
Water withdrawal	Agricultural WW	Total freshwater input, supplied from surface or groundwater, into the agriculture	Madani and Khatami (2015)
Water consumption	Agricultural WC	The portion of agricultural water withdrawal that has become unavailable for reuse due to evaporative losses, incorporation into the agricultural products, or transfer to another catchment or sea	Madani and Khatami (2015)
Water footprint	WF	A multi-dimensional indicator of water consumption which quantifies an appropriation of water resources along entire value chains. It has three components including green, blue, and gray WFs. We quantified green and blue WF components in our assessment	Hoekstra et al. (2011), Hoekstra (2019)
Green WF	${WF}_{green}$	Evapotranspiration of rainwater; volume of rainwater consumed for crop production during its lifecycle. It is quantified by dividing green ET by crop yield. We used AquaCrop-simulated yield and ET for quantifying green WFs	Hoekstra et al. (2011), Madani and Khatami (2015), Hoekstra (2019)
Blue WF	${WF}_{blue}$	evapotranspiration from surface or groundwater applied through irrigation; volume of freshwater, supplied from surface or groundwater, consumed for crop production. We used AquaCrop-simulated yield and ET for quantifying blue WFs	Hoekstra et al. (2011), Madani and Khatami (2015), Hoekstra (2019)
Blue water availability	Blue WA	Natural runoff minus environmental flow requirement (EFR). EFR is estimated based on Richter et al. (2012), as a constant fraction of natural runoff	Hoekstra et al. (2011)
WF benchmark	WF benchmark	Minimum attainable WF in the location and time that the considered crop is grown. We obtained the climate-specific benchmark levels for the considered crops from Karandish et al. (2018)	
Inefficient blue WF	$IE.{WF}_{blue}$	Inefficient blue water consumption occurs when the crop’s water footprint goes beyond its benchmark level	Hoekstra et al. (2011)
Unsustainable blue WF	$US.{WF}_{blue}$	blue WF minus blue water availability	Hoekstra et al. (2011)
Blue water scarcity	BWS	It is an indicator of water scarcity which compares actual blue WF with the sustainable one. Different BWS classes are defined by Mekonnen and Hoekstra (2016), which are summarized in Table 3	Mekonnen and Hoekstra (2016)
Attainable yield	*Y*_*att*_	The best yield achieved through skillful se of the best available technology under a given climate condition. Here, we quantified attainable yield per crop per climatic region. While differentiating between attainable rainfed and irrigated yield, our focus in the current study is on irrigated crops. maximum attainable yields were determined based on long-term local statistics	FAO and DWFI (2015)
Actual Yield	*Y*_*act*_	It reflects the current stage of soil and climate, average skill of the farmers, and their average use of technology. Actual yields are reported by local organizations	FAO and DWFI (2015)
Yield gap	YG	Yield gap is defined as different between two levels of yield and therefore, different yield gaps could be defied (FAO and DWFI 2015). Here, and following earlier researchers, we defined yield gap as the different between attainable yield and actual yield. This yield gap could be removed with improving technologies and agricultural practices. Yield gap might be even larger if we quantify it is the difference between theoretical or potential yield and actual yield	FAO and DWFI (2015)

### Study area

**Fig. 1:**
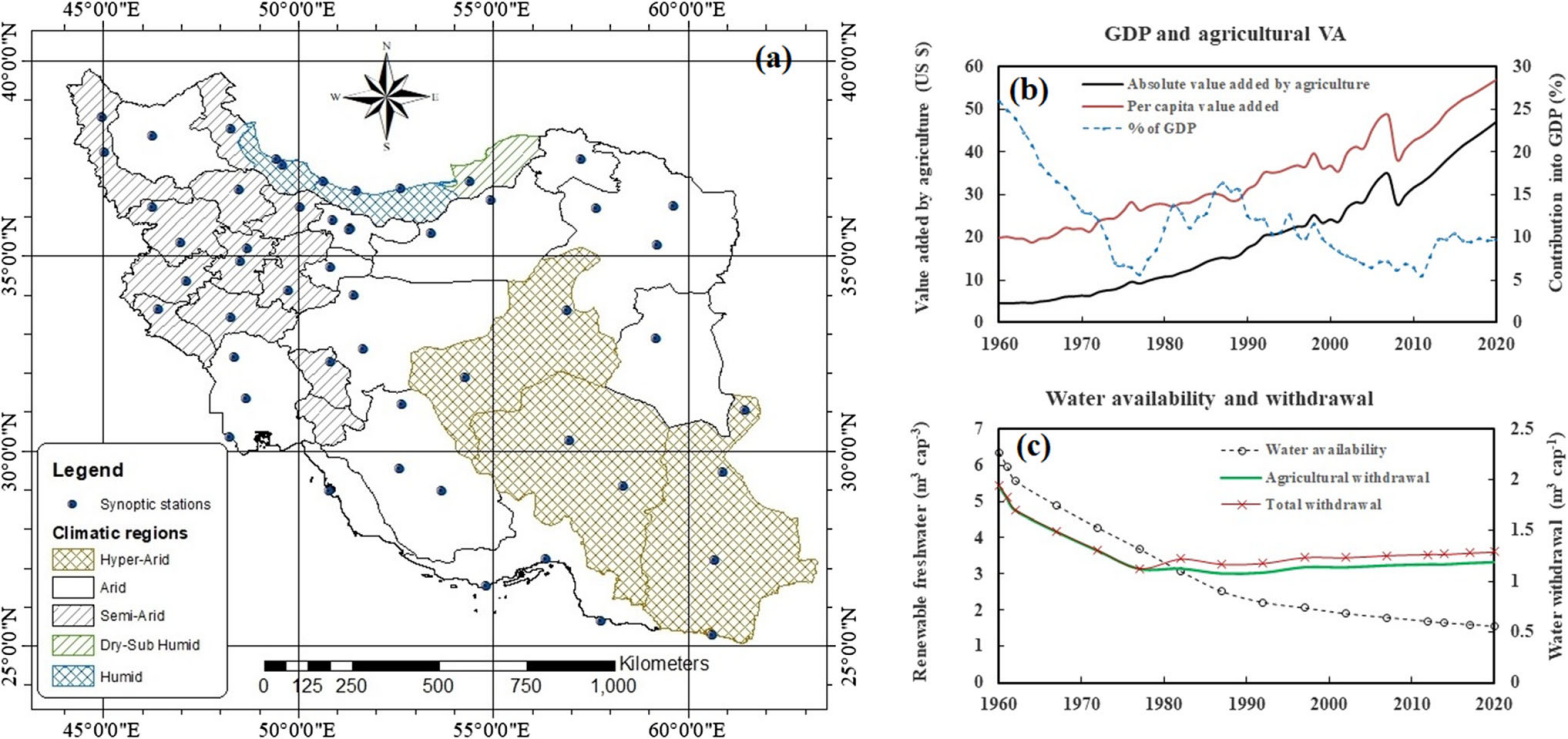
The location of the study area (**a**), the temporal variation of gross domestic production (GDP) and agricultural value added (VA) (**b**), and per capita renewable freshwater resources and withdrawal (**c**) over the period 1960–2020

This research was carried as a case study for Iran (Fig. 1), which is the second-largest country in the MENA region, and its per capita blue water availability dropped from 6350 in 1960, to 1553 in 2020. Based on the De Martonne climate classification method and using the meteorological data reported for 1985–2015, Iran is classified into five climatic zones including hyper-arid, arid, semi-arid, humid, and dry sub-humid (Fig. 1a). In 1960, 33% of the renewable blue water was withdrawn for human consumption purposes and was consumed in the agricultural, domestic, and industrial sectors. Along with a 268% increase in population, this ratio reached 68% in 2020. Agriculture is the biggest freshwater user in Iran (AQUASTAT 2020). Since 1970, and along with implementing national policies for achieving self-sufficiency in food production, the irrigated croplands and consequently, blue water consumption in agriculture considerably increased (Madani 2014). Nevertheless, only 5–16% of Iran’s gross domestic production (GDP) was supplied from the agriculture during 1979–2020 (Fig. 1b; UN 2020; WorldBank 2020), although having a contribution of 92% in gross blue water withdrawal, and 97% in net blue water abstraction (Fig. 1c; Karandish and Hoekstra 2017). Low added value obtained per drop of water consumption well indicates the low water productivity in agriculture which is also confirmed by previous researchers (Karandish and Hoekstra 2017; Nazari et al. 2018).

In this research, the status of the unsustainability and inefficiency of blue water consumed by 27 major crops grown in Iran were assessed either for the current condition or for the scenarios introduced in Sect. 2.3. These crops were classified into 8 crop categories: cereals, root and tubers, sugar crops, pulses, nuts, oil crops, vegetables, and fruits. The study period covers 10 years during 2005–2015. The 10-year average contribution of different crop categories into the national production and harvested area is summarized in Table 2 for five climatic zones of Iran; all numbers are in percentage (%). While having a contribution of 76% in total harvested area, cereals contribute 40% in national production, which indicates their relatively lower yields. Arid and semi-arid regions have the most contribution in total cereals production.

**Table 2:** The 10-year average contribution of different crop categories in national harvested area and production in different climatic zones over the study period 2005–2015. i.e., all numbers are in percentage (%)

Parameter	Crop categories	Climatic zones					Iran
		Hyper-arid	Arid	Semi-arid	Dry sub-humid	Humid	
Contribution in harvested area (%)	Cereals	51.4	75.6	80.1	75.1	66.3	75.8
	Root and Tuber	1.3	1.6	1.5	1.7	0.7	1.5
	Sugar crops	0.4	2.8	0.6	0	0	1.5
	Pulses	1.5	5.2	10.1	0.8	3.7	6.7
	Nuts	27.1	4.8	2.7	0	4.3	4.7
	Oil crops	0.5	0.8	0.9	20.5	13	2.5
	Vegetables	1.6	3.1	0.8	1.2	0.9	1.9
	Fruits	16.1	6	3.2	0.6	11.1	5.3
Contribution in total production (%)	Cereals	30.1	32.2	51.9	61.8	50	39.6
	Root and Tuber	5	6.9	13	9.8	1.5	8.1
	Sugar crops	2.1	28.4	7.5	0	0.1	18.6
	Pulses	0.4	0.9	3.1	0.2	0.7	1.4
	Nuts	14.5	1.3	1.8	0	0.9	2
	Oil crops	0.2	0.4	0.6	15.1	4	1.2
	Vegetables	7.1	18.3	8.1	10	2.5	13.7
	Fruits	40.6	11.6	13.9	3	40.3	15.3

### Estimating WFs of crop production

All calculations were done per crop, per province and per year over the period 2005–2015. Crops blue and green WFs were, respectively, estimated by dividing crop’s seasonal green and blue water consumption by crop’s yield. The green and blue water consumptions were estimated on a daily basis by distinguishing crop’s total evapotranspiration (ET) into the green and blue fractions. Crop’s ET was simulated by using the AquaCrop model (Steduto et al. 2009). The model simulates soil water balance components on a daily scale. The simulated components were then used to calculate green and blue water consumption following the procedure introduced by Hoekstra (2019). Seasonal green/blue water consumptions were then calculated by aggregating the daily values over the crop’s growing cycle.

Total green or blue water consumed within a specific province per month was then estimated by the following equations:

1 $$\left( {{\text{WF}}_{{{\text{green}}}} } \right)_{{\text{p, m}}} = \mathop \sum \limits_{i = 1}^{n} \left( {{\text{U}}{\text{.WF}}_{{{\text{green}}}} } \right)_{{\text{i,p,m}}} \times {\text{TP}}_{{\text{i,p}}}$$

2 $$\left( {{\text{WF}}_{{{\text{blue}}}} } \right)_{{\text{p, m}}} = \mathop \sum \limits_{i = 1}^{n} \left( {{\text{U}}{\text{.WF}}_{{{\text{blue}}}} } \right)_{{\text{i,p,m}}} \times {\text{TP}}_{{\text{i,p}}}$$

where, (WF_green_)_p,m_ and (WF_blue_)_p.m_ are absolute green and blue WFs (m^3^ month^−1^) consumed by all crops grown in province *p* and in month *m*, U.WF_green_ and U.WF_blue_ are unit green and blue WFs (m^3^ t^−1^), ${TP}_{i,p,m}$ is total production (*t* year^−1^) of crop *i* in province *p*, and n is the number of the crops.

All required data for the study period of 2005–2015 were collected per crop per province per year. Daily weather data were obtained from observations at 52 synoptic stations spread out over the country. Reference evapotranspiration (ETo) was then calculated based on the FAO-Penman–Monteith equation (Allen et al. 1998). Soil physical properties were extracted from Batjes (2012), and its ware parameters were obtained from the manual of the AquaCrop model (Steduto et al. 2012). Farming information, including crop’s harvest area (ha), and yield (*t* ha^−1^), planting/harvesting dates, and farming practices were obtained from Iran's Ministry of Agriculture Jihad (IMAJ 2019).

### Unsustainable/inefficient blue water consumption

*Unsustainability*. Per province per month, blue water scarcity (${\text{BWS}}_{{\text{p,m}}}$) was determined by Eq. 3 (Hoekstra et al. 2011).

3 $${\text{BWS}}_{{\text{p,m}}} = \frac{{\left( {\text{blue WC}} \right)_{{\text{p,m}}} }}{{\left( {{\text{BW}}_{{{\text{available}}}} } \right)_{{\text{p,m}}} }} = \frac{{\left( {\text{blue water consumption}} \right)_{{\text{p,m}}} }}{{{\text{NR}}_{{\text{p,m}}} - {\text{EFR}}_{{\text{p,m}}} }}$$

where ${\text{blue WC}}$ is total blue water consumption (m^3^ month^−1^), ${\text{BW}}_{{{\text{available}}}}$ is blue water availability (m^3^ month^−1^), $NR$ is natural runoff (m^3^ month^−1^), $EFR$ is environmental flow requirement (m^3^ month^−1^), *p* denotes province, and *m* denotes month. Data on monthly $NR$ per province were obtained from Iran’s Water Resource Management Company (IWRMC 2020). Following Richter et al. (2012), EFRs were assumed as 80% of natural runoffs. The BWS classes are defined in Table 3 (Mekonnen and Hoekstra 2016).

**Table 3:** Different blue water scarcity (BWS) classes defined by Mekonnen and Hoekstra (2016). A hotspot is a month/place in which blue water consumption exceeds blue water availability

Range of BWS	Scarcity status	Descriptions	Hotspot?
< 1	No scarcity	Blue water consumption is less than the available blue water	No
1–1.5	Moderate scarcity	Blue water consumption exceeded the available blue water	Yes
1.5–2	Significant scarcity	Blue water consumption exceeded the available blue water	Yes
> 2	Sever scarcity	Blue water consumption exceeded the available blue water	Yes

A month/place with a BWS > 1 is called an environmental hotspot, in which part of the blue water consumption which is beyond blue water availability, is unsuitable. In the hotspot, the unsustainable fraction of the blue WF of a specific crop will be determined based on the ratio of the blue WF of that crop to total blue water consumed in the considered hotspot:

4 $$\left( {{\text{US}}{\text{.WF}}_{{{\text{blue}}}} } \right)_{{\text{c,p,m}}} = \frac{{\left( {{\text{WF}}_{{{\text{blue}}}} } \right)_{{\text{c,p,m}}} }}{{\left( {{\text{WF}}_{{{\text{blue}}}} } \right)_{{\text{p,m}}} }} \times \left( {\left( {{\text{WF}}_{{{\text{blue}}}} } \right)_{{\text{p,m}}} - \left( {{\text{BW}}_{{{\text{available}}}} } \right)_{{\text{p,m}}} } \right)$$

where, $\left( {{\text{US}}{\text{.WF}}_{{{\text{blue}}}} } \right)_{{\text{c,p,m}}}$ is the unsustainable blue water footprint for crop *c* in province *p* and month *m*, $\left( {{\text{WF}}_{{{\text{blue}}}} } \right)_{{\text{p,m}}}$ and (WF_blue_)_c,p,m_ are, respectively, total and the considered crop’s blue WF in province *p* and month *m*, and (BW_available_)_p,m_ is local blue water availability in province *p* and month *m*.

*Inefficiency*. Crop’s blue water consumption is inefficient when it goes beyond its benchmark levels. We obtained the climate-specific benchmark levels for the considered crops from Karandish et al. (2018). Per crop, per province, and per month, the inefficient blue WF was then determined as the difference between actual and benchmark WFs of the considered crops.

### Scenario assessment and consequences

As summarized in Table 4, a total of 15 alternative management scenarios compared to the current (field/irrigation) management were defined by considering the individual or combined effects of the following measures:

- Yield gap closure
- Replacing current irrigation systems with drip irrigation
- Soil mulching with organic matters
- Applying deficit irrigation by reducing irrigation water depth by 15%

**Table 4:** The 15 alternative management scenarios defined compared to the current on-farm management in the study area

Scenario no.*	Name of scenario	Drip irrigation (DRIP)	Soil mulching (MULCH)	Deficit irrigation (DI15%)	Yield gap closure (YGC)
1	DR	*			
2	MU		*		
3	DI			*	
4	YGC				*
5	DR.MU	*	*		
6	DR.DI	*		*	
7	DR.YGC	*			*
8	DR.MU.DI	*	*	*	
9	DR.MU.YGC	*	*		*
10	DR.DI.YGC	*		*	*
11	MU.DI		*	*	
12	MU.YGC		*		*
13	MU.DI.YGC		*	*	*
14	DI.YGC			*	*
15	DR.MU.DI.YGC	*	*	*	*

^*^Drip irrigation is applied for all crops, except for wheat, barley, and rice, for which this irrigation technique is not technically feasible. Soil mulching was assumed to be applied within all croplands unless rice fields, since rice is irrigated by basin method; hence, soil mulching is not relevant. In deficit irrigation, crops were assumed to receive 15% less blue water in each irrigation event during their entire growing periods. Yield gaps were calculated per crop per climatic zone while differentiating between the irrigated and rainfed crops

In yield gap closure scenario, the climate-specific attainable yields for a specific crop and in a specific climatic zone was assumed to be the upper quantile (top 25%) of yield statistics reported for that crop in the considered zone over the study period 2005–2015. Yield gap was then estimated as the difference between actual and attainable yields.

In Drip irrigation scenario, the existing irrigation system is assumed to be replaced by drip irrigation system except for rice, wheat and barley, for which applying drip irrigation is not technically feasible in the study area.

Soil mulching was assumed to be applied within all croplands unless rice fields, since rice is irrigated by basin method; hence, soil mulching is not relevant. In deficit irrigation scenario, crops were assumed to receive 15% less blue water in each irrigation event during their entire growing periods. The other 11 combined scenarios were defined as different combinations of these four measures.

The environmental consequences of all scenarios were then assessed in terms of their effects on blue water consumption and saving, unsustainable/inefficient fraction of blue WFs, and BWS. These consequences were compared with the relevant values under the current condition, which is called the base case in this research. Among 15 defined scenario, the best scenario was then selected as one which has the largest effect on reducing unsustainable and inefficient blue WF and BWS. We name it as “the best scenario” afterward.

Finally, the socioeconomic consequences were assessed for the best alternative management scenario in terms of its effects on the daily number farm-employees (NFE), provincial food security (FSp), the fraction of international food export (IFE), and gross domestic production (GDP). Per crop per province, Iran's Ministry of Agriculture Jihad (IMAJ) defined standard numbers for NFE per unit of crop production (cap t^−1^) (IMAJ 2019). These values were used to estimate NFE for the base case and for the best scenario. Per province, FSp is defined by dividing local food production by local food demand. Local food demand is estimated by multiplying per capita food demand by local population. International food export is estimated as surplus production beyond national demand. The IFE was then calculated by dividing total export by total production. GDP for the current condition was extracted from Statistical Center of Iran (SCI 2016). For the best scenario, the value added by the additional production was added to the current GDP.

## Results

### Unsustainability/inefficiency in blue WF under current condition

**Fig. 2:**
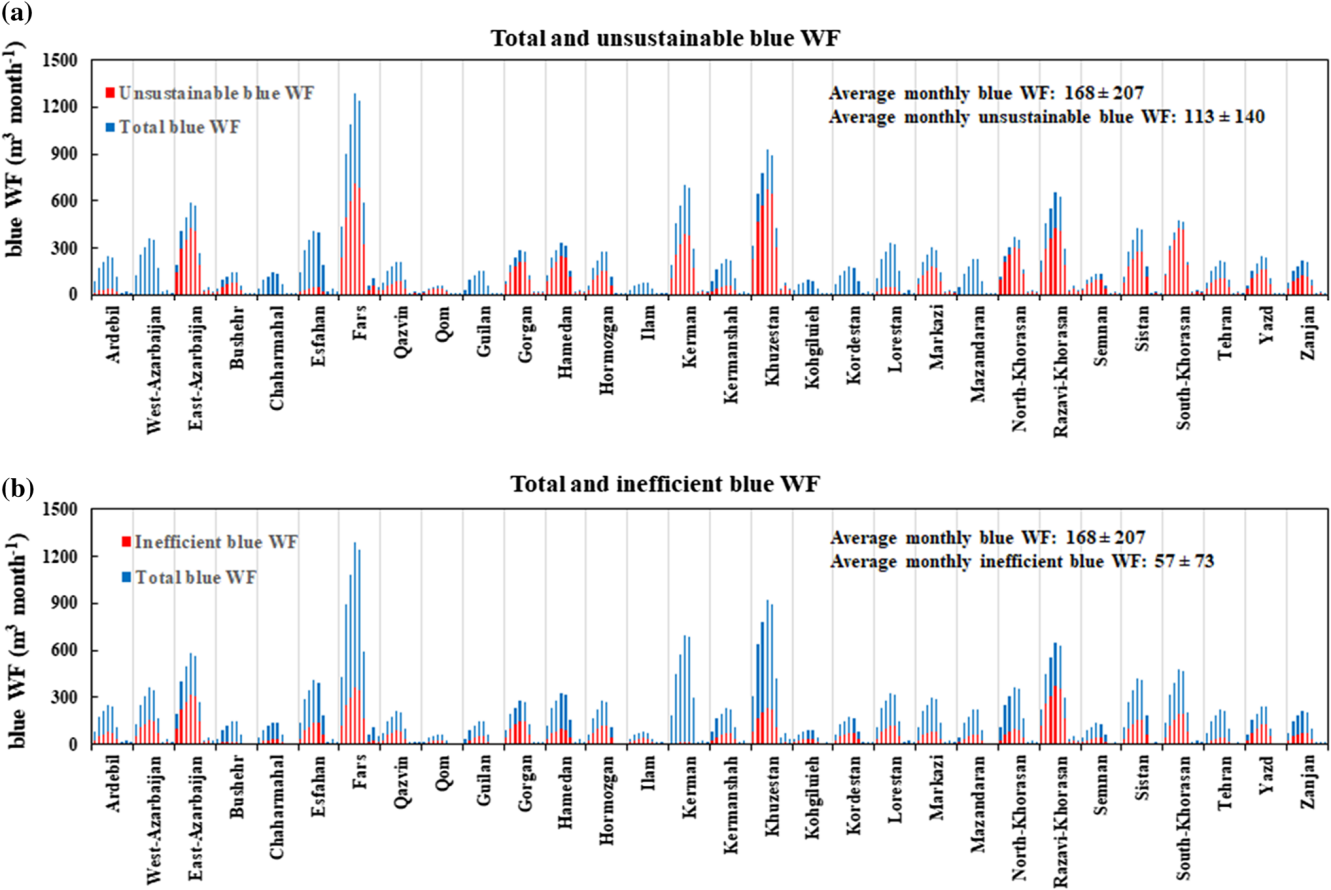
The 10-year average of monthly unsustainable (**a**) and inefficient (**b**) blue water consumption in different provinces of Iran over the study period 2005–2015. For each province, 9 months are included as vertical bars starting from April and ending at December. The vertical grids differentiate between different provinces

Annually, 182–3181 million m^3^ year^−1^ of blue water is consumed unsustainably within the hotspots (BWS > 1) (Fig. 2a). Monthly unsustainable blue water consumption occurs in 23 provinces over the period April–November; accounted for 0.92–712 million m^3^ month^−1^, which comprises 12–89% of monthly blue water consumptions in these provinces. April and October, has the less contribution in annual US.WF_blue_, while July has the most one. Three provinces of Fars, Khuzestan, and Razavi-Khorasan, locating within the arid zone with less blue water availability, have the highest monthly US.WF_blue_. In Seven provinces of Chaharmahal&Bakhtirari, Ilam, Kohgiluyeh and Boyer-Ahmad, Kordestan, and West-Azarbaijan (in the semi-arid zone), and Gilan and Mazandaran (in the humid zone), blue water is fully consumed sustainably in the agricultural sector (i.e., US.WF_blue_ = 0 and BWS < 1).

On an annual scale, 67–1661 million m^3^ year^−1^ of blue water is consumed inefficiently in different provinces (Fig. 2b). Provincial monthly inefficient blue water consumption varies in the range of 0.1–373 million m^3^ month^−1^, which comprises 3–57% of monthly blue water consumption in different provinces. December and July have the lowest and the highest monthly IE.WF_blue_, respectively. Furthermore, Kerman province has the lowest monthly IE.WF_blue_, and three provinces of Razavi-Khorasan, Fars, and East-Azarbaijan (all in the arid zone) has the highest ones.

**Fig. 3:**
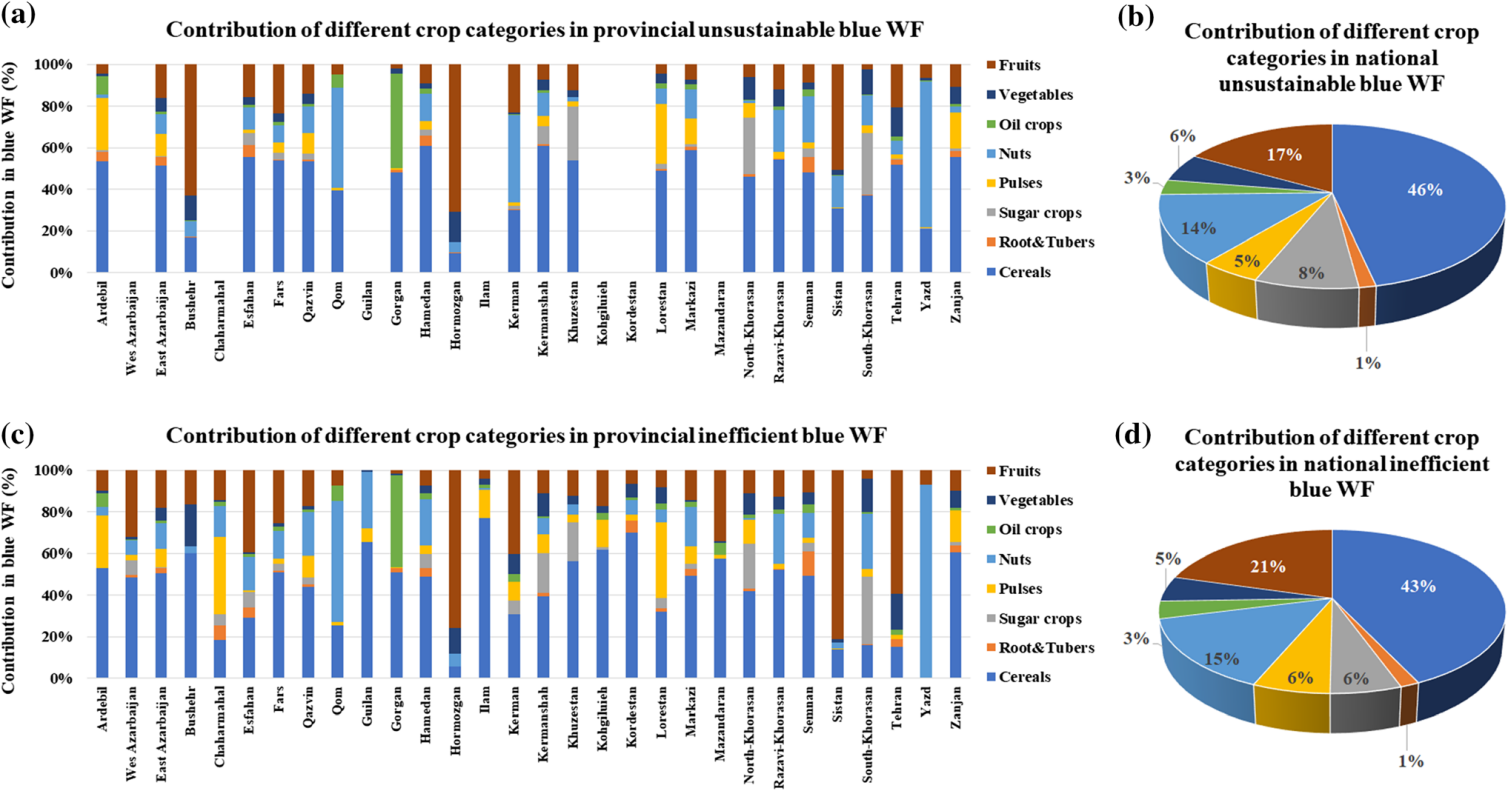
The 10-year average of the contribution of different crop categories in provincial and national unsustainable (**a**, **b**) and inefficient (**c**, **d**) blue WFs over the period 2005–2015

Figure 3 shows the 10-year average of the contribution of different crop categories in provincial and national US.WF_blue_ (Fig. 3a, b) and IE.WF_blue_ (Fig. 3c, d) overt the study period. Of 35.5 billion m^3^ year^−1^ US.WF_blue_ consumed within the entire irrigated croplands in the study area, 46%, 17%, and 14% is consumed by cereals, fruits, and nuts, respectively, while the other 23% is consumed by the other five crop categories. Cereals also had the highest contribution of 43% (6.7 billion m^3^ year^−1^) in the national IE.WF_blue_ (15.5 billion m^3^ year^−1^), followed by fruits (21%), and nuts (15%). Such a dominant contributions of these crop categories are also observed at the province scale.

### Unsustainability/inefficiency in blue WF under proposed scenarios

**Fig. 4:**
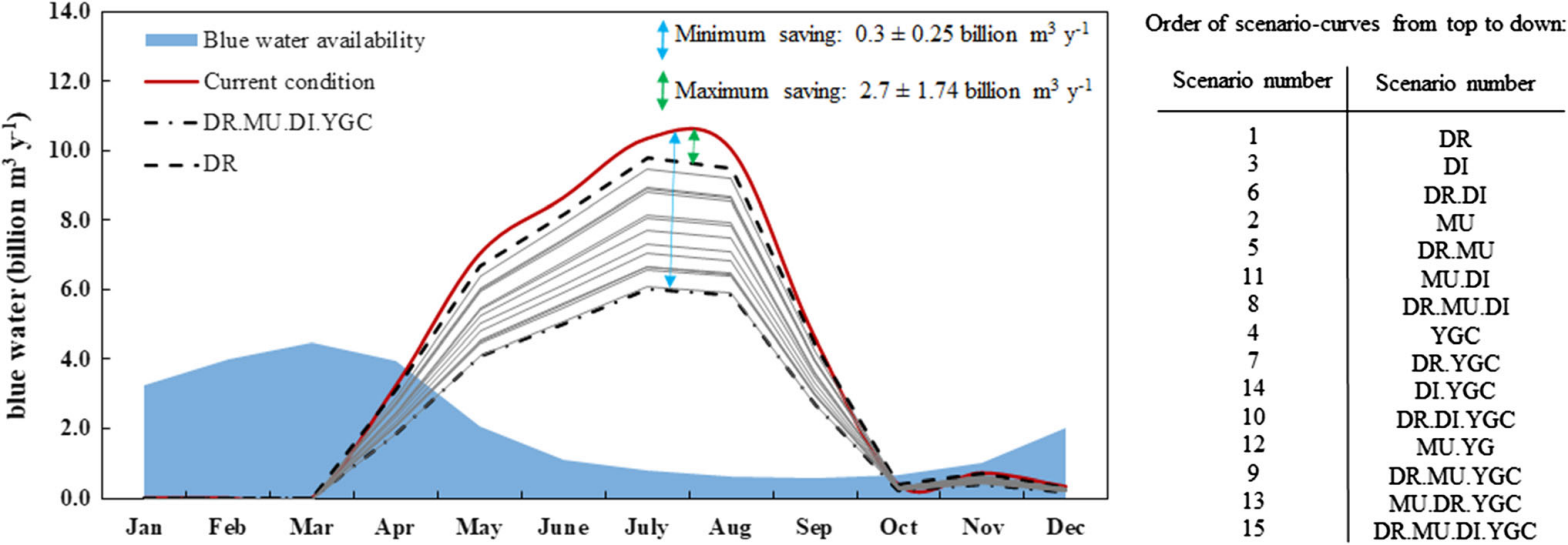
The 10-year average of monthly blue water consumption and availability under current and 15 proposed alternative scenarios over the study period 2005–2015. The scenarios with the lowest and highest blue water saving compared to base case (i.e., the current condition over the period 2005–2015) were highlighted in the figure, which were, respectively, drip irrigation scenario (DR) and the combination of yield gap closure (YGC), drip irrigation (DR), mulching (MU), and deficit irrigation applied at the rate of 15% reduction in crop’s blue water demand (DI). Table in right shows the order of curves for different management scenarios from top to down

Figure 4 shows the monthly blue water availability and consumptions under the current condition and 15 alternative management scenarios and the contribution of different crop categories in blue water saving. Compared to the current condition, all scenarios have the potential of blue water saving, and the highest savings always occur in August (accounted for 2–19 billion m^3^ month^−1^). Replacing current irrigation systems with drip irrigation when feasible (i.e., DR scenario) comes with the lowest blue water saving of 4.8 billion m^3^ year^−1^ (a 5% saving compared to the current condition). In contrast, combining yield gap closure with drip irrigation, mulching, and deficit irrigation at the rate of 15% (i.e., the DR.MU.DI.YGC scenario), will save a total of 38.3 billion m^3^ year^−1^ blue water (a 42% saving compared to the current condition). In this scenario, the largest saving occurs within the cereals field.

**Fig. 5:**
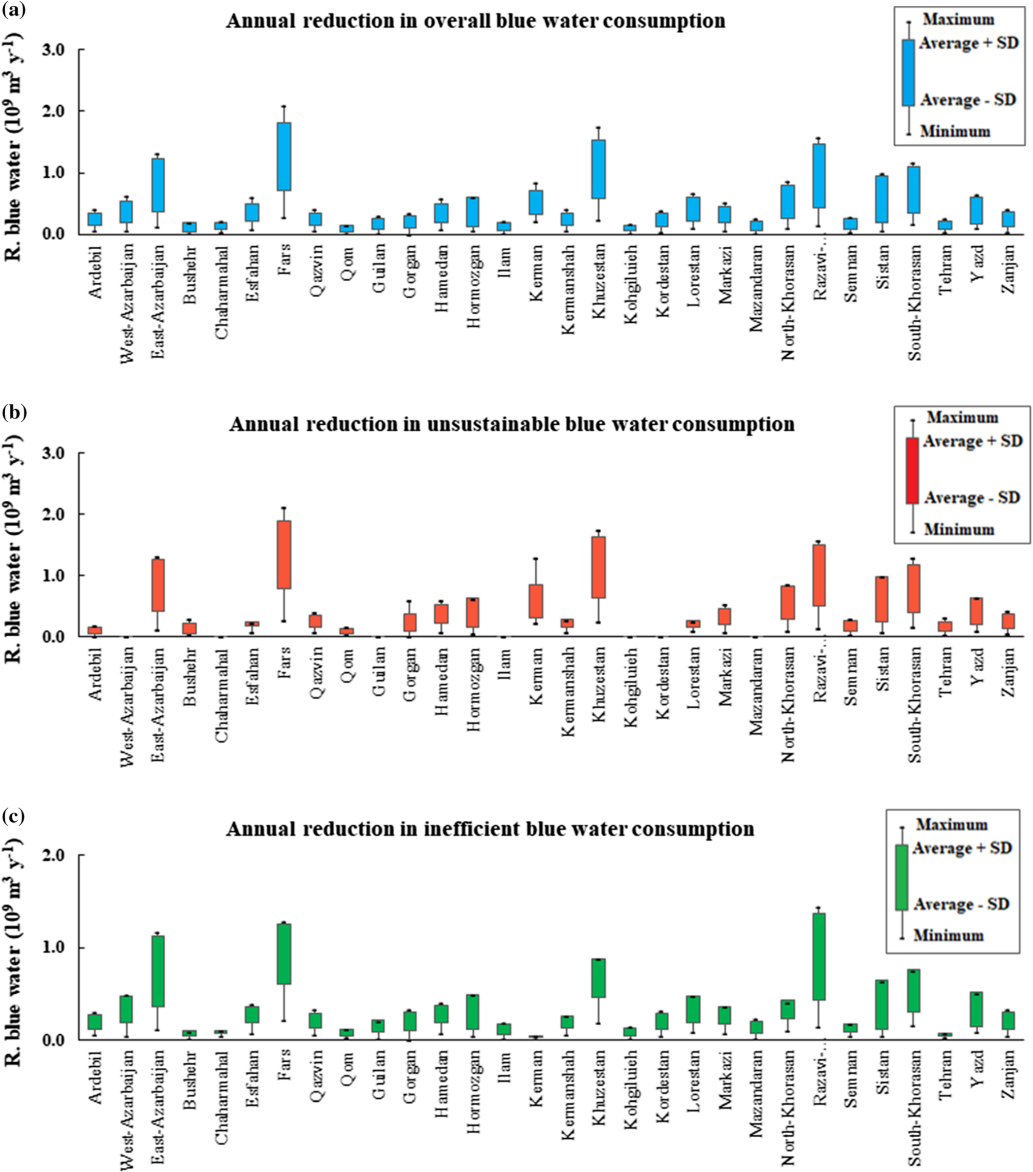
Average, minimum, and maximum reduction in annual overall (**a**), inefficient (**b**), and unsustainable (**c**) blue water consumption under 15 alternative management scenarios compared to the base case. Except for Gorgan, Mazandaran, and Hormozgan provinces, the lowest and the highest reductions in the overall, unsustainable, and inefficient blue water consumptions occur under the DR and the DR.MU.DI.YGC scenarios, respectively. For Goragn, Mazandaran, and Hormozgan provinces, the lowest and highest reductions occur under the MU and the DR.MU.DI.YGC scenarios, respectively. i.e., DR denotes drip irrigation scenario, MU denotes mulching scenario, and the DR.MU.DI.YGC denotes the scenario in which yield gap closure is combined with drip irrigation, mulching, and deficit irrigation

Figure 5 shows the degree to which these savings can reduce provincial US.WF_blue_ and IE.WF_blue_. Except for a few cases, the lowest and the highest reduction in US.WF_blue_ and IE.WF_blue_ occurs under the DR and the DR.MU.DI.YGC scenarios, respectively. Based on the ensemble averages of all scenarios, provincial US.WF_blue_ will decrease from 96 ± 45 million m^3^ year^−1^ (in Qom province) to 1337 ± 556 million m^3^ year^−1^ (in Fars province) (Fig. 5b); and the provincial IF.WF_blue_ will decrease from 32 ± 11 million m^3^ year^−1^ (in Kerman province) to 931 ± 321 million m^3^ year^−1^ (in Fars province) (Fig. 5c).

Table 5 shows the degree to which US.WF_blue_ and IE.WF_blue_ can be reduced for different crop categories. Based on the ensemble averages of all scenarios, the largest reduction in US.WF_blue_ and IE.WF_blue_ will occurs within the cereals lands, accounted for 4.6 ± 2.2 billion m^3^ year^−1^ and 4.1 ± 1.7 billion m^3^ year^−1^, respectively. This result attributes to the fact that cereals are dominant either in the national harvested area or in national crop production. The lowest reduction, however, will occur within the croplands under root and tuber (accounted for 0.14 ± 0.1 billion m^3^ year^−1^ for US.WF_blue_, and 0.13 ± 0.1 billion m^3^ year^−1^ for IE.WF_blue_). This result is caused by the fact that potato, as the only crop in root and tuber category, has the lowest blue WF, and so does the lowest unsustainable and inefficient fractions.

**Table 5:** Ensemble average, minimum, maximum, and standard deviation (SD) of the potential reductions in unsustainable (US.WF_blue_) and inefficient (IE.WF_blue_) blue WFs under the defined scenarios for different crop categories in the study area over the period 2005–2015

Crop categories	Reduction in US.WF_blue_ (billion m^3^ year^−1^)				Reduction in IE.Wfblue (billion m^3^ year^−1^)			
	Ensemble average	Minimum	Maximum	SD	Ensemble average	Minimum	Maximum	SD
Cereals	4.64	7.89	0.27	2.18	4.04	6.00	0.03	1.65
Root and Tuber	0.14	0.23	0.04	0.05	0.13	0.19	0.03	0.05
Sugar crops	0.76	1.21	0.30	0.30	0.65	0.85	0.32	0.18
Pulses	0.73	1.11	0.28	0.39	0.64	0.89	0.27	0.30
Nuts	1.98	3.14	0.59	0.98	1.56	2.25	0.48	0.77
Oil crops	0.26	0.41	0.12	0.09	0.24	0.36	0.09	0.08
Vegetables	0.56	0.85	0.23	0.29	0.50	0.72	0.21	0.24
Fruits	1.36	2.32	0.34	0.77	1.04	1.63	0.27	0.61

### Blue water scarcity alleviation

**Fig. 6:**
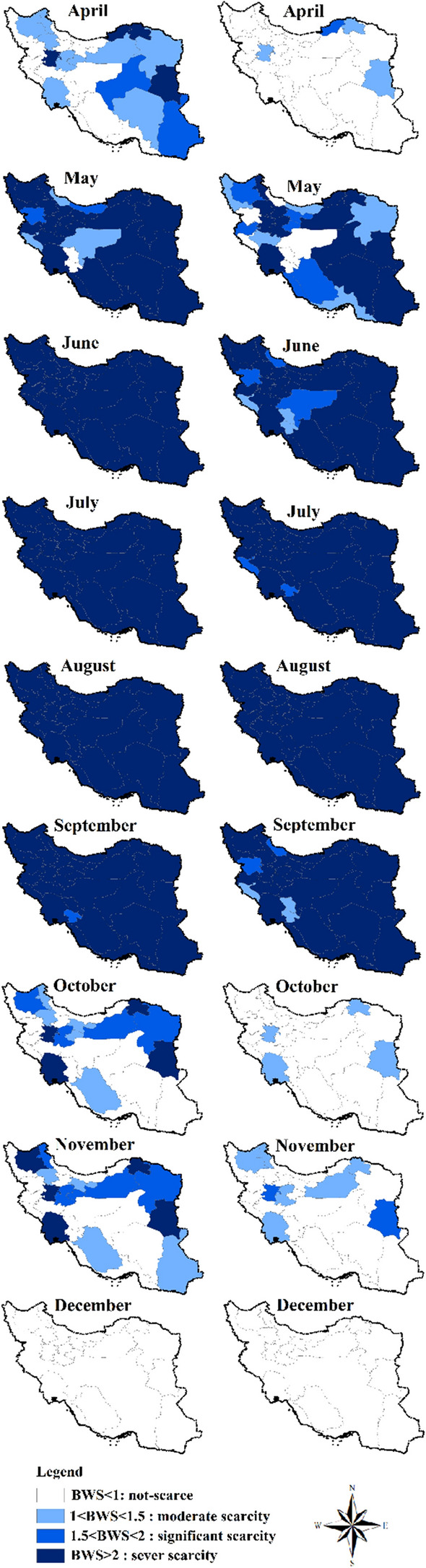
Spatial distribution of monthly blue water scarcity (BWS) under the base case (i.e., the current condition over the period 2005–2015) (maps in the left hand), the scenario with maximum blue water saving (i.e., the combination of yield gap closure (YG), drip irrigation (DRIP), mulching (MULCH), and deficit irrigation applied at the rate of 15% reduction in crop’s blue water demand (DI15%)) (maps in the left hand)

Figure 6 shows monthly BWS under the current condition and the DR.MU.DI.YGC scenario (i.e., the best scenario out of 15 defined scenarios which results in the highest reduction in US.WF_blue_ and IE.WF_blue_). For the current condition, overconsumption of blue water occurs during the April–November period at the cost of violating EFRs (BWS > 1), and possibly, using the fossil water resources. In April, moderate to significant BWS occurs in roughly half of the country, while the next half absents from BWS. In May, most of the provinces experience a sever BWS. During the July–September period, sever BWS occurs for almost all provinces since the growing periods of many crops fall within this period in the study area. However, BWS in Autumn (October and November) is low and occurs only in a few provinces.

Reduced blue water consumption under the DR.MU.DI.YGC scenario will change the status of BWS in many provinces and months. Unless for a few provinces, the overall blue water consumption remains below blue water availability (BWS < 1) during the October-December period, and in April. In May, the status of BWS will change from ‘sever’ to ‘moderate’/ ‘significant’ in ten provinces, and will change to ‘not-scarce’ in four provinces. However, the status of BWS remains unchanged in the majority of the provinces over the period June–September.

While the DR.MU.DI.YGC scenario can alleviate BWS to some extent, it is still insufficient to address the problem of blue water overconsumption in the agricultural sector. Despite of the promising outcomes, there will be many provinces and months with BWS > 1; hence, other measures should be explored in addition to those introduced in this research to solve the problem.

### Socio-economic consequences under the best measure

**Fig. 7:**
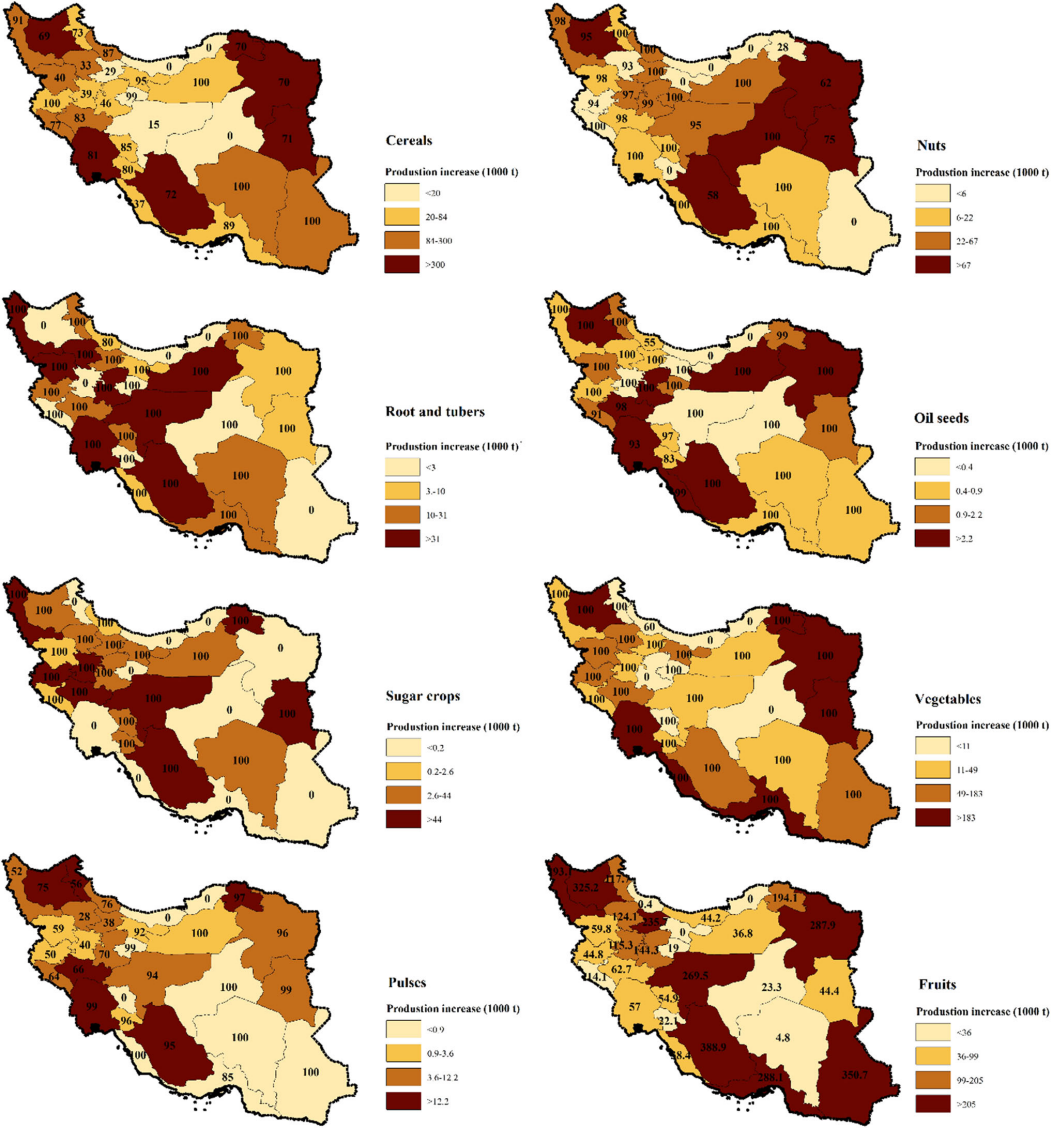
The provincial gap between the actual and climate-specific attainable yield for different crop categories in the study area over the period 2005–2015. The numbers in the maps denotes the contribution of irrigated crops in total production gap

The socioeconomic consequences of implementing the best water saving scenario among 15 defined ones (the DR.MU.DI.YGC scenario) are estimated and results are summarized in Table 6. Under the DR.MU.DI.YGC scenario, annual crop production will increase by 17.8 million t year^−1^ due to yield gap closure (87.6% compared to the current condition, Fig. 7), and blue water consumption will decrease due to applying drip irrigation, mulching, and deficit irrigation. These outcomes result in a considerable reduction in crop’s unit WFs (m^3^ t^−1^). Hence, national GDP will increase by 54%, from 21 billion US$ year^−1^ to 32.4 billion US$ year^−1^, and provincial GDP will increase by 380 ± 415 million US$ year^−1^ (Table 6). As a consequence, provincial food security will increase by 9% due to the increased contributions of different provinces in crop production. Hence, the dependency on international food import will decrease by 5%, and the contribution to international food export will increase by 87% (from 22 to 42%). The DR.MU.DI.YGC scenario has also the potential of reducing unemployment rates since the number of farm-employees will increase by 132%, from 9.1 thousand people days^−1^ to 21.1 thousand people days^−1^.

**Table 6:** Socioeconomic consequences of implementing the best water saving agricultural practices in the study area. i.e., the best scenario refers to the combination of fulfilling yield gap (YG), drip irrigation (DRIP), mulching (MULCH), and deficit irrigation applied at the rate of 15% reduction in crop’s blue water demand (DI15%)

Parameter	Base case	The best scenario	Average increase (%)
Provincial GDP (million US$ y^−1^)	700 ± 511	1080 ± 789	54
National GDP (million US$ y^−1^)	21 002	32 390	54
Provincial FS (%)	53 ± 18	58 ± 18	9
Contribution in international export (%)	22	42	87
International import dependency (%)	2.7	2.6	− 5
Number of employee per day (1000 people)	9.1	21.1	132

## Discussion

In this study, we tried to map out the WF scheme of the agricultural sector in Iran, and propose pathways for alleviating blue water scarcity in the country. This study includes various limitations and uncertainties. First, the AquaCrop model was used to estimated ET and yield while using the default parameters in some cases. When data are available, calibrating and validating the model against field conditions will provide more accurate results. Second, the use of data with different spatial resolutions may cause some inconsistencies in the given results. In addition, the results may be sensitive to different environmental conditions; for instance, the results may change when hydrologically differentiating between the dry and wet periods (Zhuo et al. 2016), or when more detailed equations are applied to estimate EFRs (Karandish et al., in press). Hence, their impacts can be assessed in further investigations.

Our monthly WF assessment indicates that over the period 2005–2015, Iran consumed 0.17 ± 0.21 million m^3^ month^−1^ of its blue water resources for crop production, 113 ± 40 million m^3^ month^−1^ of which was consumed unsustainably within hotspots (Fig. 2a), and 57 ± 73 million m^3^ month^−1^ consumed inefficiently (Fig. 2b). The spatial variation of the unsustainable and inefficient blue WFs is partly due to the spatial variation of crops’ ET, which depends on seasonal and inter-annual climatic status change (Khazaei et al. 2019). Iran has a large geographic diversity of hydro-climatic regimes due to having a spatial difference of 16.65 °C for its annual average temperature, and 1107 mm y^−1^ for its annual precipitation (Madani 2014; Moshir Panahi et al. 2020). The site-specific behavior of unsustainable and inefficient blue WFs in Iran is also caused by the regional differences of cropping patterns.

The unsustainable and inefficient water consumption in Iran’s agricultural sector roots in different reasons. First, the country is suffering from a natural water scarcity. Over the past decades, Iran became warmer, its annual precipitation and surface runoff experienced a decreasing trend, and faced with severe droughts in 1990 and 2008 (Karandish et al. 2017; Moshir-Panahi et al. 2020). For instance, annual precipitation in the western basins, which are the backbone of crop production in Iran (Karandish et al. 2017), decreased by 65 mm year^−1^ over the period 1986–2016 (Moshir-Panahi et al. 2020). Continuing agriculture production under such condition entailed exacerbating unsustainable exploitation of the limited blue water resources. Recently, Moshir-Panahi et al. (2020) also reported that Iran’s current evapotranspiration is beyond its sustainable level which could be maintained by only annual renewable water input.

Second, Iran’s technical capacity is limited by different economic and political sanctions, which caused the lack of access to novel technologies, international scientific exchanges, and foreign investments. Hence, the lack of industrialized agriculture and using almost outdated farming technologies and practices is among the major causes of inefficient agriculture in Iran (Madani 2014; Madani et al. 2016).

Third, while climate variability, climate change, droughts, and sanctions exposed undeniable impacts on Iran’s water resources, its water challenges in the agricultural sector is mostly man-human-made.

Maghrebi et al. (2020) reported the insensitivity of irrigated crop productions to natural water availability changes in the water-scarce regions of Iran, which reveals that the historical development of the irrigated agriculture in Iran was not compatible with its blue water availability changes. Over the period 1981–2013, total irrigated production in Iran increased by 137% (Maghrebi et al. 2020), while the local statistics show that total water shortage changes (including surface and groundwater) followed a decreasing trend of 16 mm y^−1^ in this period, which implies a considerable decline in Iran’s blue water availability (Moshir Panahi et al. 2020). On the other hand, a considerable part of irrigated farming expansions took place in the southern, eastern, and central parts of the country, with higher reliance on groundwater resources (Maghrebi et al. 2020). The expansion of irrigated croplands in Iran was motivated by the national food policy implemented in 1979 to achieve self-sufficiency in food production (Madani 2014; Karandish and Hoekstra 2017). The increased blue water demand was then fulfilled through an extensive building of artificial reservoirs (Madani 2014), overexploitation of groundwater resources (Mirzaei et al. 2019; Karandish et al. 2020), and implementation of inter-basin water transfer projects (Mirchi and Madani 2016; Karandish et al. 2021). Indeed, developing irrigated agriculture occurred at the cost of consuming the non-renewable blue water resources which have been supplied through building 127 dams with an overall storage capacity of about 35 billion m^3^ year^−1^, and drilling more than 540 000 wells across the country (Maghrebi et al. 2020). In other words, Iran’s decision makers tried to solve Iran’s water scarcity by increasing water supply through infrastructure development rather than doing strict water conservation efforts (Madani et al. 2016). Hence, the long-term continuation of overlooking the linkage between environment and development planning caused serious water problems which may last for generations (Darzi-Naftchali et al. 2020).

Our scenario assessment revealed that Iran’s blue water scarcity could be moderated through modifying agricultural practices and water management. The combination of YGC with drip irrigation, mulching, and deficit irrigation (i.e., the DR.MU.DI.YGC scenario) has the largest water saving potential. In this scenario, YGC has the largest effect on reducing unsustainable and inefficient blue WFs. Earlier researchers also indicated the key role of yield improvement in reducing crop’s WFs (Zhuo et al. 2014). While Rosa et al. (2018) indicated that Iran’s yield gap could be closed through expanding irrigation infrastructures into the rainfed lands, we didn’t focus on water-limited yield gap in the current research. The main reason is that Rosa et al. (2018) carried out their BWS assessment on an annual scale and for the country as whole, which hides the intra-annual and intra-national variabilities in blue water availability and demands. Our finer spatial/temporal resolution BWS assessment well reveals that the majority of the country experiences moderate to severe blue water scarcity over the period May–September. Hence, YGC through replacing rainfed lands with irrigated one should be done by caution and required further deeper assessments of the consequences before being implemented. Indeed, improving rainfed/irrigated crop’s yield to the maximum climate-specific one, while keeping water consumption unchanged, should be of higher priority. Such yield benchmark levels may be achievable in Iran since a majority of the croplands are suffering from the lack of input managements, best available technologies and proper agricultural practices (Mueller et al. 2012; Rosa et al. 2020). However, further research is also required to assess the extent to which yield gap could be closed without increasing unsustainable and inefficient blue water consumption.

In addition to improving the environmental status, implementing the DR.MU.DI.YGC scenario will also improve the national socioeconomic status by decreasing unemployment rate and the dependency on international food import, and by increasing GDP, food security level, and the contribution to international export. Reduced unemployment rate and increased GDP will prosper the welfare of the inhabitants, particularly in the rural area. In addition, increased local food security will increase the resilience to the unexpected international shocks, such as market shocks or pandemic diseases. Nowadays, the power is going to be transferred from the large agricultural companies and global markets to the local actors (DuPuis and Goodman 2005; Burnett and Murphy 2014). Kinnunen et al. (2020) stated that localness of food systems is the main source of many narratives associated with sustainability. They indicated that closing crop’s yield gap and reducing food losses are among two key drivers which promote the stability of eating local.

However, the current study considered the benefits of yield gap closure, drip irrigation, mulching, and deficit irrigation but not the cost. Practically, the beneficial effects of applying these measures might outweigh their costs. Hence, the costs of these measures for reducing BWS should be evaluated by using a marginal cost assessment like one carried out by Chukalla et al. (2017). In addition, the feasibility of applying these measures vary from crop to crop and place to place; therefore, further researches are required to evaluate the stability of the proposed solutions (Read et al. 2014).

Furthermore, our monthly BWS reveals that while the DR.MU.DI.YGC scenario has the potential of reducing the number of hotspots (i.e., place/month with BWS > 1, Fig. 6), there are still many cases in which crop production takes place at the cost of violating EFRs. Nouri et al. (2019) also reported that a combination of soil mulching with drip irrigation couldn’t fully address monthly blue water scarcity in the Upper Litani Basin. Our results show that EFR violations mainly occur during the dry periods, while there is no BWS over the wet period of December-March; when the natural runoff is far beyond the EFRs. Hence, a plausible solution to address water shortage in such hotspots is to store addition water in the artificial reservoirs during the wet periods, and to release them during the dry periods. Zhuo et al. (2019) demonstrated that water footprint cap in the dry periods, when crop’s water demand is the most, could be raised when runoff variability is smoothed by constructing reservoirs along the river in the Yellow-River basin in China. Nouri et al. (2019) also emphasized on the positive role of artificial reservoir on reducing BWS during the dry periods. However, dam constructions may also have negative socioeconomic and environmental consequences. For instance, Zhuo et al. (2019) stated that when water storage in the wet periods goes beyond 20% of natural runoff, then the EFR wouldn’t be met in this period, which results in off-season BWS. On the other hand, reservoirs themselves are considered as blue water consumers. The overall evaporated water from reservoirs surfaces equals to 25% of total blue water annually consumed by irrigated agriculture/municipal/industrial sectors in the world (Hogeboom et al. 2018; Mekonnen and Hoekstra 2012). Hence, this solution also requires further comprehensive assessments before being implemented.

## Conclusion

Fulfilling the increasing food demand requires the sustainable and efficient use of finite water resources. This study assesses the possibilities for reducing unsustainability and inefficiency in crop’s blue water consumption while preventing food security losses. The study was carried out as a case study for Iran, where the available water consumption pattern involves 78% and 34% unsustainability and inefficiency, respectively, resulting in moderate to severe blue water scarcity (BWS) in a large part of the country over the period April–November. Hence sustaining agriculture in this country requires substantial adjustments to current technologies and agricultural practices and water management. In this regard, blue water saving possibilities were assessed through defining 15 alternative management scenarios compared to the base case by considering the individual or combined effects of four major measures: yield gap closure, drip irrigation, soil mulching, and deficit irrigation. Results show that all scenarios have positive impacts on reducing unsustainability and inefficiency in blue water consumption nationwide, while combining all four mentioned measures, with a potential blue water saving of 19 million m^3^ year^−1^, has the largest effect. Such a potential water saving reduces unsustainable and inefficient blue water consumption by 48% and 84%, respectively; environmental deterioration is also expected to be lowered since this scenario has the potential of releasing monthly BWS or changing its status to the smoother levels in up to 11 provinces. Moreover, the socioeconomic condition may also improve along with an increase of 132% in the daily number of farm-employees, 9% in provincial food security, 87% in international food export contribution, and 54% in gross domestic production. While the findings of this study confirm that the sound and sensible agriculture through the defined scenarios have the potentials of contributing to food and water security with lower impacts on natural ecosystems, but by far they are not sufficient to fully address BWS in many provinces. Indeed, orienting the current food production system to a more sustainable trajectory requires exploring other measures such as conservation tillage, cultivating different crop cultivars, or modifying the cropping pattern. Besides, the findings of the current research show the potential of reducing BWS in the dry period by storing additional water in artificial reservoirs during not-scarce wet periods. However, dam constructions have their own consequences and need to be deeply investigated before implementation. If none of these measures have the potential of solving blue water overconsumption, then the harvested area under the irrigated crops should be reduced.
